# Immunogenic self-peptides - the great unknowns in autoimmunity: Identifying T-cell epitopes driving the autoimmune response in autoimmune diseases

**DOI:** 10.3389/fimmu.2022.1097871

**Published:** 2023-01-09

**Authors:** Jörg Christoph Prinz

**Affiliations:** Department of Dermatology, University Clinics, Ludwig-Maximilian-University of Munich, Munich, Germany

**Keywords:** autoimmunity, HLA association, immunopeptidome, T cell receptor (TCR), polyspecificity, T-cell epitope, immunogenic self-peptides, psoriais

## Abstract

HLA-associated autoimmune diseases likely arise from T-cell-mediated autoimmune responses against certain self-peptides from the broad HLA-presented immunopeptidomes. The limited knowledge of the autoimmune target peptides has so far compromised the basic understanding of autoimmune pathogenesis. This is due to the complexity of antigen processing and presentation as well as the polyspecificity of T-cell receptors (TCRs), which pose high methodological challenges on the discovery of immunogenic self-peptides. HLA-class I molecules present peptides to CD8^+^ T cells primarily derived from cytoplasmic proteins. Therefore, HLA-class I-restricted autoimmune responses should be directed against target cells expressing the corresponding parental protein. In HLA-class II-associated diseases, the origin of immunogenic peptides is not pre-specified, because peptides presented by HLA-class II molecules to CD4^+^ T cells may originate from both extracellular and cellular self-proteins. The different origins of HLA-class I and class II presented peptides determine the respective strategy for the discovery of immunogenic self-peptides in approaches based on the TCRs isolated from clonally expanded pathogenic T cells. Both involve identifying the respective restricting HLA allele as well as determining the recognition motif of the TCR under investigation by peptide library screening, which is required to search for homologous immunogenic self-peptides. In HLA-class I-associated autoimmune diseases, identification of the target cells allows for defining the restricting HLA allotype from the 6 different HLA-class I alleles of the individual HLA haplotype. It furthermore limits the search for immunogenic self-peptides to the transcriptome or immunopeptidome of the target cells, although neoepitopes generated by peptide splicing or translational errors may complicate identification. In HLA class II-associated autoimmune diseases, the lack of a defined target cell and differential antigen processing in different antigen-presenting cells complicate identification of the HLA restriction of autoreactive TCRs from CD4^+^ T cells. To avoid that all corresponding HLA-class II allotypes have to be included in the peptide discovery, autoantigens defined by autoantibodies can guide the search for immunogenic self-peptides presented by the respective HLA-class II risk allele. The objective of this article is to highlight important aspects to be considered in the discovery of immunogenic self-peptides in autoimmune diseases.

## 1 Introduction

Autoimmune diseases are a group of immunologically mediated diseases in which autoreactive lymphocytes evade tolerance and are activated against the body’s own structures. Depending on the functional differentiation of the autoreactive T cells and the formation of autoantibodies, this leads to organ-specific (ankylosing spondylitis, type I diabetes, autoimmune thyreoiditis, multiple sclerosis etc.) or systemic (e.g. Behçet’s disease, systemic lupus erythematodes etc.) tissue damage, chronic inflammation with tissue remodeling (psoriasis vulgaris etc.) or to functional changes (myastenia gravis, Graves’ disease etc.).

Genome-wide association studies (GWAS) have shown that the immune-mediated inflammatory diseases considered autoimmune to date have a complex genetic predisposition. While many of the associated risk gene loci or gene variants are pleiotropic, i.e., associated with different diseases, association with specific HLA-class I and/or HLA-class II alleles mediates the actual disease-specific risk (1). In part, the association relates to single nucleotide polymorphisms that affect the binding of the peptide antigen to HLA, thereby altering the repertoire of antigens presented to T cells. The respective HLA alleles were initially identified as candidate risk genes. For several common immune-mediated diseases, GWAS have confirmed these associations in large patient and control subject cohorts and, as in the case of psoriasis, have provided *de facto* evidence by sequencing the complete HLA region (2, 3). The HLA association of autoimmune diseases is considered to indicate that disease-associated HLA allotypes mediate an autoimmune response against certain self-peptides of the immunopeptidome, as different HLA molecules present different peptide repertoires to T cells.

Prime examples for primarily HLA-class II-associated autoimmune diseases are rheumatoid arthritis (4, 5), systemic lupus erythematosus (6), pemphigus vulgaris (7) and bullous pemphigoid (8), and celiac disease (9). Multiple sclerosis (10) and type I diabetes mellitus (11) show associations with both HLA-class I and class II alleles. Distinct HLA-class I alleles with a strong associations with autoimmune diseases are HLA-B*27 that is associated with ankylosing spondylitis (12), HLA-C*06:02 with psoriasis (13), and HLA-B*51 with Behçet’s disease (14). In each of the HLA-class I-associated diseases, disease risk is controlled by variants in the gene encoding endoplasmic reticulum aminopeptidase 1 (ERAP1). The respective HLA-associations are summarized by Matzaraki et al. (1).

In this article I will discuss key aspects that I consider relevant to the identification of the immunogenic self-peptides of autoreactive T-cell responses in autoimmune diseases. Special aspects incorporate my own experience from the identification of target cell and autoantigen of the autoimmune response in psoriasis. The article is not intended as an overview of the methodological approaches, which have recently been summarized in detail (15).

## 2 HLA-alleles, T-cell antigen recognition and immunopeptidomes

### 2.1 Which are the pathogenic T cells?

HLA molecules present peptides for recognition by TCRs of T cells. Antigen processing and presentation pathways do not distinguish between self- and foreign proteins. Accordingly, the immunopeptidomes presented by HLA molecules consist predominantly of self-peptides. T cell activation against these peptides is prevented by central and peripheral tolerance mechanisms [reviewed in (16)]. Identification of the immunogenic self-peptides that stimulate the autoimmune response requires knowledge of the autoreactive T-cell populations. T cells respond to the recognition of peptide epitopes with clonal proliferation. Clonal T-cell expansion in the inflammatory infiltrate of autoimmune tissue damage thus indicates selection and activation by locally presented self-peptides regardless of whether it is an organ-specific autoimmune response such as in type I diabetes (11) or a systemic autoimmune disease such as systemic sclerosis (17). The HLA association of the respective disease may designate the actual pathogenetically relevant T-cell subpopulation. HLA-class I molecules present peptides to CD8^+^ T cells. Therefore, CD8^+^ T cells likely drive autoimmune pathology in HLA-class I-associated autoimmune diseases. This is supported by dominant clonal expansions of CD8^+^ T cells in the inflammatory infiltrate in ankylosing spondylitis, psoriasis and psoriatic arthritis (18–20) and by the prominent infiltration of CD8^+^ T cells in vasculitis lesions of Behçet’s disease (21). According to the antigen presentation of HLA-class II molecules to CD4^+^ T cells, CD4^+^ T cells should by highly relevant for the autoimmune pathology in HLA-class II-associated diseases. Again, their clonal expansion in the infiltrate is an indication that they mediate tissue damage. In addition, CD4^+^ T cells may help B cells in inflamed tissue and drive the formation of autoantibodies (22). Genetic associations with both HLA-class I and class II risk alleles as well as clonal expansions of both CD4^+^ and CD8^+^ T cells in the inflammatory infiltrate of type I diabetes (11), however, complicate the decision as to which T-cell populations are ultimately pathogenetically critical and should be addressed to investigate T-cell epitopes, because they are likely to interact in the autoimmune process. While in type 1 diabetes mellitus the HLA-class II alleles mediate the main genetic risk (1, 11), HLA-A*02:01, which is also associated with the disease, leads to an autoimmune reaction of CD8^+^ T cells against a peptide from the insulin B chain and β cell destruction (23).

### 2.2 Autoantibodies, seronegative diseases and the immunological synapse

Autoimmune diseases associated with HLA-class II alleles are often characterized by autoantibody profiles that are specific for the respective disease, thus making the character as an autoimmune disease obvious. Furthermore, the antigens recognized by the autoantibodies can be screened for HLA-class II presented epitopes that activate CD4^+^ T cells. HLA-class I-mediated diseases, in contrast, are seronegative. Here, it is assumed that the autoimmune reaction is mediated directly by CD8^+^ T cells. For seronegative HLA-class I-associated diseases, autoimmune pathogenesis is often controversially discussed because the evidence for an autoimmune response is mostly indirect. It consists in the therapeutic efficacy of immunosuppressive drugs or of antibodies blocking T cell-specific or T cell-relevant cytokines. Identification of clonal or oligoclonal T-cell expansions, defined as groups of T cells sharing the CDR3 α- and CDR3 β-chain amino acid sequences, as well as conserved TCR patterns of CD8^+^ T cells in tissue lesions of autoimmune diseases such as psoriasis, psoriatic arthritis, ankylosing spondylitis or multiple sclerosis (18–20, 24–28), support that autoimmune T-cell activation is driven by disease-specific autoantigens which may be identical in different patients. In contrast to the autoantibodies in HLA-class II-associated autoimmune diseases, which allow direct detection of autoantigens by immuno-serological methods, targets of the T-cell response in seronegative diseases are usually not obvious. Therefore, their character as an autoimmune disease can ultimately only be proven by the unequivocal identification of the autoantigens leading to T-cell activation.

Specific activation of T cells occurs in protective and pathogenic immune responses through the immunological synapse (29). It consists of the presenting HLA molecule, peptide antigen and TCR. TCR and cognate HLA molecule are required for the identification of a T-cell antigen. Advances in the analysis of peptides presented by HLA molecules and the mechanisms underlying antigen recognition by TCRs during the last decade have provided a completely new understanding of autoimmune responses. In the following, I will explain the three components of the immunological synapse with respect to the presentation and TCR recognition of self-peptides. I will then address essential aspects that are important in the identification of immunogenic self-peptides in autoimmune diseases.

### 2.3 Presentation of peptides by classical HLA-class I molecules directs immune responses against target cells

HLA-class I molecules present peptides derived from proteins expressed within the cytoplasm to CD8^+^ T cells. Accordingly, they direct a CD8^+^ T cell-mediated immune response against target cells expressing the parental protein of the antigenic peptide. This circumstance, which in principle serves the recognition of intracellular pathogens and mutations in malignant cell transformations (30), is a basic prerequisite for the understanding of HLA-class I-mediated autoimmune diseases.

HLA-class I molecules consist of an allotype-specific heavy chain and the invariant β2-microglobulin light chain. The HLA-class I heavy chains are encoded on the short arm of chromosome 6 in region p21.1-21.3 by three different loci: HLA-A, HLA-B and HLA-C. HLA-class I molecules are expressed on almost all nucleated cells as well as on platelets. The HLA-class I alleles are highly polymorphic. By the year 2019, nearly 20,000 different HLA-class I alleles had been identified (31). Much of the polymorphisms are located in Exon 2 and 3 of the HLA-class I allele and cause non-synonymous amino acid changes. They encode the α1 and α2 domains that determine peptide binding by specifying the amino acids at specific positions of the bound peptides that allow their anchoring in the pockets of the binding groove. The HLA-class I-presented peptides result from proteins that have been degraded by the cellular 26S proteasome in the cytoplasm (30). Immunological stimuli such as IFN-γ integrate additional proteolytic subunits into the proteasome, increasing its activity and expanding the spectrum and number of peptides generated (32, 33). This allows the immunoproteasome to process a larger substrate pool for presentation under inflammatory or infectious conditions. Approximately one third of the presented peptides are subject to NH_2_-terminal trimming of elongated precursor peptides to the length required for binding into the peptide binding groove by two endoplasmic reticulum aminopeptidases, ERAP1 or ERAP2. However, both enzymes can also destroy corresponding peptides and thus withdraw them from presentation (34–37).

Peptides are selected for presentation by the respective HLA alleles based on the anchor amino acids. Accordingly, each of the different allelic products presents its own peptide repertoire (38–40). The interval between anchor residues limits the minimum length of the presented peptides to 8 amino acids, since shorter peptides lack sufficient anchors (41). The total length of the presented peptides is usually limited to 10 amino acids because the binding groove of HLA-class I molecules is closed on both sides. Bulging of peptides may allow the presentation of longer peptides up to a length of ~14 amino acids (42). The mode of peptide binding allows the presentation of more than one million different peptides by each HLA allotype.

### 2.4 Principles of peptide presentation by classical HLA-class II molecules by professional antigen-presenting cells

HLA-class II molecules present antigens to CD4^+^ cells (30). They are heterodimers that are encoded by three polymorphic loci: HLA-DR, HLA-DP and HLA-DQ. HLA-DQ and -DP molecules are composed of polymorphic α- and β-chains. For HLA-DR, a non-polymorphic α-chain combines with a polymorphic β-chain encoded by the HLA-DRB gene. Most HLA-DR haplotypes express a HLA-DRB1 gene as well as a second HLA-DRB3, -DRB4, or -DRB5 gene that both combine as heterodimers with HLA-DRα. A heterozygous human thus expresses 6 or 8 pairs of HLA-class II α- and β-chain molecules, one pair each of DP and DQ, and one or two pairs of DR molecules. HLA-class II molecules are mainly expressed by professional antigen-presenting cells (APCs). They include dendritic cells, monocytes and macrophages, and B cells. However, non-professional APCs such as keratinocytes or melanocytes can also express HLA-class II molecules under inflammatory conditions (43). HLA-class II-molecules scan both the environment and cytosol. Extracellular molecules are taken up through the endo-lysosomal pathway (44, 45). In this process, phagosomes fuse with lysosomes for the formation of phagolysosomes for protein degradation by the lysosomal proteases. In autophagy organelles, mitochondria, ribosomes or small amounts of the cytosol are encapsulated in autophagosomes, which fuse with endosomes to introduce cytoplasmic and nuclear molecules into the HLA-class II presentation pathway (45–47). Various tissue-specific processing pathways and antigen-presenting cell types may further increase the spectrum and origin of epitopes presented by HLA class II (48). HLA-class II molecules are also highly polymorphic, and the polymorphisms determine differences in the peptide binding groove. It includes a series of pockets that determine the side chains of amino acid residues of the nonameric core of the presented peptides (49, 50). Since the HLA-class II peptide binding groove is open on both sides, peptides binding to class II molecules tend to be of variable length typically between 13 and 25 residues. This implies much less selection pressure for presentation, as the peptides do not have to match a predetermined length, and generates a broad and hardly predictable spectrum of peptides derived from extra- and intracellular proteins. In this respect, the immunopeptidomes of HLA-class II molecules should potentially exhibit much greater diversity than those of HLA-class I molecules.

### 2.5 HLA immunopeptidomes mainly originate from self-proteins

The set of peptides presented by an HLA allotype is referred to as the HLA peptidome, HLA ligandome, or immunopeptidome. The progress in mass spectrometry technology has facilitated detailed characterization of the immunopeptidomes eluted from HLA-class I and HLA-class II molecules. Here, HLA-peptide complexes are isolated from detergent solubilized lysates by immunoaffinity purification, followed by extraction and purification of the HLA-bound peptides. Peptide spectra obtained by tandem liquid chromatography-mass spectrometry are aligned with the spectra of peptides from protein sequence databases (51). Limitations in the analysis exist for peptides that are too hydrophobic or too hydrophilic, that may be incompatible with ionization. Without addressing further details or methodological differences, the analyses of numerous cell lines show that the immunopeptidomes of both HLA-class I and HLA-class II molecules contain thousands of different peptides from endogenous proteins (47, 52, 53).

More than half of all proteins expressed in a cell type may be represented in HLA-class I immunopeptidomes, and up to 98% of all peptides isolated from HLA-class I and class II molecules of different cell lines originated from self-proteins (53, 54). Cross-presentation, in which both MHC molecules intersect intracellular and extracellular pathways, increases the diversity of HLA-class I and class II immunopeptidomes (45). Evaluation of the peptides isolated from the different allotypes has also provided accurate information regarding the peptide binding motifs specific to each HLA allotype. Interestingly, the HLA-class I alleles HLA-C*06:02, HLA-C*07:01, HLA-C*07:02, HLA-C*07:04, and HLA-B*27 associated with psoriasis are clustered in the same HLA supertype due to overlapping anchor residues in the peptides (51, 55), thus potentially presenting the same psoriasis-specific immunogenic self-peptides.

Neo-epitopes may be generated by translational errors (56). The immunopeptidome repertoire is furthermore extended by various posttranslational modifications, including proteasomal splicing of peptide fragments (57–59). Spliced peptides are nonlinearly templated peptides that are assembled during proteasomal digestion by cis-splicing from different regions of the same protein or by trans-splicing from two peptides of different proteins. The proportion of spliced peptides differs between different HLA allotypes. Especially for HLA-B*27:05 associated with ankylosing spondylitis, more than 40% of the eluted peptides were cis- or trans-spliced products. Peptides eluted from HLA-B*51, the main risk allele of Behçet’s disease, accounted for approximately 25% of the HLA-B*51 peptidome. Spliced peptides broaden the peptidome spectra (57, 59, 60), although their contribution to the total immunopeptidome is highly controversial and may be overestimated due to methodological artifacts (61–63).

The non-linearly templated peptides increase the diversity of target peptides for recognition by T cells and possibly create a particular risk of escaping tolerance mechanisms and triggering autoimmunity. Three types of peptides therefore need to be considered for the identification of immunogenic HLA-presented self-peptides of autoimmune reactions: peptides with true linear sequences > nonlinearly templated peptides including spliced peptides > untemplated peptides with no current biological explanation (64).

### 2.6 TCRs are polyspecific

T cells recognize the complex of HLA molecule and antigenic peptide by means of their TCR. Mature T cells express unique heterodimeric TCRs consisting of a TCRα- and a TCRβ-chain (αβ TCRs), each of which contributes to antigen recognition. The TCR chains are generated by random rearrangement of different gene segments. The TCR α-chain is formed by somatic recombination of one of approximately 43-45 different variable-region genes (*TRAV*) with one of 50 joining gene segments (*TRJA*) and the gene for the constant region of the α-chain (*TRAC*). For the β-chain, one of 40-48 variable region genes (*TRBV*) is linked to one of 2 diversity (*TRBD*) and one of 12-13 joining (*TRBJ*) gene segments and the gene for the constant region of the β-chain (*TRBC*) (65). The junctional diversity of the chains is expanded by random nucleotide insertions and deletions at the rearrangement sites. This generates the *de novo* diversity in the complementarity determining region 3 (CDR3) of each chain contacting the antigenic peptide in the MHC binding cavity. The CDR1 and CDR2 encoded within the *TRAV* and *TRBV* genes contact the MHC molecule. All 3 CDRs from both the α- and β-chain mediate TCR docking to the peptide/MHC interface. Random pairing of different TCR α- and TCR β-chains generates a huge combinatorial diversity of the TCR repertoire that exceeds 10^20^ αβ-TCRs (66). However, the actual human repertoire is much smaller with approximately 10^11^ unique TCRs (67), although estimations of the actual repertoire size are strongly influenced by the applied repertoire profiling technique and differ between studies (68). Regardless of the precise size, the spectrum of possible HLA-presented peptides greatly exceeds the human TCR repertoire. To cover all possible antigens without a hole in the antigenic spectrum, TCRs must be polyspecific (69). Polyspecificity is due to the antigen recognition mode of TCRs. Unlike antibodies, which are specific for a particular epitope, TCRs are ligated by antigenic peptides that display their respective peptide recognition motif. It is defined by two or three amino acids anchoring the peptides in the discrete pockets of the peptide binding groove of the cognate HLA molecule, and one or two amino acids contacting the TCR, while tolerating a broad amino acid diversity at the other positions of the antigenic nonamer core sequence (70, 71). Estimates proposed that a single TCR may potentially recognize up to 10^4^-10^7^ different MHC-associated epitopes (72).

## 3 Identification of TCR epitopes in HLA-associated autoimmune diseases

The challenge in deciphering autoimmune responses is to specifically identify the particular self-peptide(s) of the immunopeptidome that become immunogenic during lifetime and cause activation of autoreactive T cells. This is equally true for peptides from the HLA-class I and HLA-class II immunopeptidome. For HLA-class II-associated autoimmune diseases, the autoantibodies can contribute to the identification of epitopes in the corresponding autoantigens recognized by T cells, which control autoantibody production in B cells. For HLA-class I-mediated autoimmune responses, only the TCR is available for this purpose.

The tremendous progress in the knowledge of immunopeptidomes may require a more precise terminology. The clonal selection theory had stated that one lymphocyte recognizes one antigen (73). We now recognise that the HLA immunopeptidomes consist of thousands of different self-peptides, a few of which trigger autoreactive T cell activation during life through the ligation of a polyspecific TCR contained in the preexisting T cell pool. The term “antigen” originally referred to substances that gen(erate) the formation of anti(bodies). In a T cell-mediated autoimmune response, however, individual self-peptides from the complex immunopeptidome become immunogenic by inducing a T cell-mediated immune response. Therefore, in the following I will try to use the terms “immunogenic” or “epitopes” for self-peptides that elicit T cell-mediated immune responses.

### 3.1 Identification of self-epitopes of HLA-class I-restricted autoimmune responses

Proof of an autoimmune disease requires the identification of the respective self-peptides that activates the T cell-mediated autoimmune response. Current approaches to identify T cell epitopes use different strategies. They are based on staining T cells with recombinant peptide-loaded major histocompatibility complex (MHC) multimers, on selecting phage particles, yeast or insect cells displaying pMHC complexes in large libraries with immobilized or soluble recombinant TCRs, or activating recombinant TCRs in cell-based systems by coculture with APCs cotransfected with HLA and combinatorial peptide libraries (15, 70, 74–81). A critical prerequisite is identification of the pathogenic TCRs and the restricting MHC allotype. Following recombinant expression the TCRs are being used to identify peptide ligands presented from peptide libraries by the corresponding MHC molecules. The amino acid sequences of these mimotopes, which mimic the actual immunogenic self-peptide, then allow to determine the particular TCR recognition motif used to search for proteins with homologous peptide sequences. Ultimately, the actual significance of candidate peptides for the autoimmune response must then be proven.

#### 3.1.1 Main strategy to identify self-epitopes of autoimmune T-cell responses

- Identification of clonally expanded and thus presumably pathogenic CD4^+^ or CD8^+^ T cells by single cell TCR analysis (see section 3.2).
- Cloning and functional expression of the cDNA of the paired α- and β-chains of clonal TCRs (see section 3.3).
- For HLA-class I-restricted autoimmune responses, identification of the target cell and determination of HLA restriction using the recombinant TCRs (see section 3.4).
- Characterization of the peptide recognition motif of the TCRs of interest by peptide library screening (see section 3.5).
- Screening the transcriptome or immunopeptidome of the target cell, or databases of the human proteome for proteins that contain peptide sequences corresponding to the peptide recognition motif (see section 3.5, 3.6).
- Evaluation of the immunogenicity of these self-peptides for the TCR of interest (see section 3.6).
- Analyzing the immunogenicity of candidate self-peptides under natural conditions (see section 3.6).

### 3.2 Identification of αβ TCRs of clonally expanded T cells

Identification of pathogenic T cells requires detection of clonally expanded CD8^+^ T cells by single cell αβ-TCR analysis. For this purpose, T cells must be recovered from the inflammatory infiltrate. This can be accomplished by harvesting live cells from fresh tissue samples or laser dissection from tissue sections. To examine the immune pathogenesis of psoriasis, we had formerly developed a multiplex PCR, which for the first time allowed amplification, cloning, and sequencing of the cDNA of the α- and β-TCR chains of individual T cells isolated by micropipetting from explant culture (19). Today, paired αβ-TCR chains of individual T cells can be determined by next generation high-throughput single-cell sequencing and transcriptomic analysis of complex T cell populations using PCR-based barcoding strategies and others (82, 83).

For the identification of immunogenic self-peptides, it is essential to capture the T cells that are pathogenetically relevant. In HLA-class I-associated autoimmune diseases these are the CD8^+^ T cells, for HLA-class II-associated autoimmune pathology CD4^+^ T cells. Furthermore, the correct tissue localisation of the T cell infiltrate must be considered. HLA-C*06:02-associated psoriasis, for example, results from the recruitment, activation, and expansion of CD8^+^ T cells in lesional epidermis (19, 84), whereas the bulk of the inflammatory infiltrate, consisting of predominantly irrelevant CD4^+^ T cells lacking signs of specific expansion, is localized in the underlying dermis. Accordingly, TCR analysis of the total infiltrate from full-skin biopsies covering dermis and epidermis failed to identify clonally expanded T cells (85). In conditions such as ankylosing spondyloarthritis it may be necessary to distinguish between infiltrating T cells in the synovial tissue and those in the joint effusion (28).

### 3.3 Need for unlimited TCR availability

Although various tumor antigens have been identified using T cell lines, the short-lived nature of *in vitro* expanded CD8^+^ T cells limits their use in searching for immunogenic self-peptides in autoimmune diseases. Here, recombinant expression systems with unlimited availability of the specific TCRs are required. For this purpose, the cDNA of the paired α- and β-chains of the TCRs of interest must be cloned and functionally expressed in accordance with the peptide library system chosen. Different strategies employed cellular or cell-free expression systems. As an example, TCR multimers were generated by complexing C-terminally biotinylated alloreactive mouse TCRs of known specificity with streptavidin labeled with phycoerythrin. In a cell free system devoid of coreceptors, staining of peptide (p)MHC libraries expressed in yeast with these multimers allowed for enrichment of sets of individual clones from the libraries by fluorescence activated sorting. The encoded peptides revealed shared TCR recognition motifs with the actual antigen, although none of the library peptides corresesponded to known proteins (86). Nevertheless, this approach had clearly proven that peptide libraries can be used to identify the particular recognition motif of TCRs for further searching for immunogenic peptides.

In the case of psoriasis, we had employed a cell-based platform to define the specificity of lesional psoriatic TCRs (80, 87, 88). TCR α- and β-chains of clonally expanded lesional psoriatic CD8^+^ T cells were cotransfected together with CD3, human CD8 α and β chains and super green fluorescent protein (sGFP) controlled by nuclear factor of activated T cells into the 58α^-^β^-^ mouse T hybridoma reporter cell line, which lacks endogenous TCR α- and β-chains. Accordingly, these TCR hybridomas carry the specificity of the lesional autoimmune response. They indicate specific TCR ligation by the induction of sGFP.

### 3.4 Target cell identification of the autoimmune response: Key to identify the restricting HLA-class I allotype and the immunogenic self-peptide

Most strategies to establish methods for T-cell epitope identification employed TCRs with known specificity and MHC restriction. From the identified peptides, they concluded that the identification of unknown T cell epitopes is possible using the respective approaches. The search for immunogenic self-peptides in autoimmune diseases, however, is confronted with additional challenges:

- In autoimmune diseases, the immunogenic self-peptides are unknown and the presenting HLA allele is not defined. Even though the disease risk allele specifies the HLA allotype likely mediating the autoimmune response, the HLA restriction of the examined TCRs must be determined from the six different HLA-A, HLA-B, or HLA-C alleles of the respective individual HLA haplotype.
- In line with polyspecificity, it is likely that a TCR may recognize several self-peptides from different proteins. It must be verified that candidate epitopes are actually processed from the parental protein by the cell’s proteasome to exactly fit into the peptide binding groove for HLA-class I presentation.
- Due to the limited size of peptide libraries relative to all possible MHC peptides, it is unlikely that the peptide eliciting the autoimmune response of interest will be directly identified by peptide library screening.

Identification of the autoimmune target cell expressing the parental protein of the immunogenic self-epitope may solve these issues. The reactivity of TCRs with target cells expressing a defined HLA haplotype can be used to define the restricting HLA allele. It further narrows the origin of HLA-class I-presented epitopes to the cellular proteome or the immunopeptidome of the target cells, allowing verification of the identified candidate peptides as disease-specific immunogenic self-peptides.

Target cell identification may be achieved through different approaches. One possibility is to stain tissue cells with fluorescent TCR multimers (89). We had co-cultured TCR hybridomas generated from lesional psoriatic CD8^+^ T cell clones with skin-specific cell types, which corresponded to the actual site of the immune response. A pathogenic psoriatic Vα3S1/Vβ13S1-TCR was selectively activated by HLA-C*06:02-positive primary melanocytes and melanoma cell lines, defining both the HLA restriction and the target cell of the psoriatic autoimmune response for further examination (88).

### 3.5 Defining the peptide recognition motif of TCRs by peptide library screening

Completely randomized peptide libraries have a much lower efficacy in identifying potential T-cell epitopes. If the anchor residues for the cognate HLA allotype are known, introducing limited diversity at these positions can maximize the number of peptides that can be correctly displayed by the respective MHC molecule. We had used both completely randomized combinatorial peptide libraries and peptide libraries with predetermined anchor positions for HLA-C*06:02 to identify mimotopes of the psoriatic Vα3S1/Vβ13S1 TCR. They showed overlapping amino acid sequences (88). In general, the identification of TCR ligands requires a repeated enrichment or subcloning of the isolated library peptide clones. Regardless of which strategy is used to identify TCR epitopes by the peptide libraries, the amino acids at each position of the identified peptide ligands are subsequently compiled into heat maps and translated into sequence logos that define the TCR recognition motif. The TCR motif is then applied to searches for proteins containing corresponding peptide sequences in the cellular proteome, in databases using various search algorithms, or in the corresponding immunopeptidome. In the case of nonlinearly encoded peptides, analysis of the immunopeptidome of the respective target cell would be required. However, this is often not available, and its determination requires high target cell numbers, which may be difficult to generate. The immunogenicity of the candidate peptides identified in this way must then be verified by TCR ligation.

### 3.6 Evidence for the autoimmune role of self-peptides must be provided in the context of the parental protein and target cell

For the identification of immunogenic self-peptides further aspects have to be considered. According to polyspecificity, it can be expected that several epitopes in multiple proteins are identified as TCR ligands. The peptides must be excised from the parental protein to the length required for presentation. Therefore, it is necessary to demonstrate their immunogenicity under “natural conditions”. The key step for the transformation of a protein into HLA-class I–restricted epitopes is usually processing by the proteasome (90). While the cellular proteasome basically generates the C-terminus, some of the presented peptides additionally require NH_2_-terminal trimming by endoplasmic reticulum aminopeptidases to the length for presentation (35, 36). For the psoriatic autoimmune response, we had initially identified 6 peptides from human proteins through homology search that were presented by the psoriasis risk allotype, HLA-C*06:02, and stimulated the pathogenic psoriatic Vα3S1/Vβ13S1 TCR (88). Of the six Vα3S1/Vβ13S1 TCR ligands, only the peptide from ADAMTS-like protein 5 (ADAMTSL5) could be processed into its immunogenic form from the parental protein, whereas the other peptides were not immunogenic in the context of full-length proteins. Knock down in melanocytes and mutational analysis of ADAMTSL5 further confirmed the immunogenicity of the ADAMTSL5 peptide. Furthermore, only melanocytes as target cells of the Vα3S1/Vβ13S1 TCR were able to generate the immunogenic peptide from the parental ADAMTSL5, whereas full-length ADAMTSL5 was not immunogenic in other cell types (88). Generation of the antigenic ADAMTSL5 peptide in melanocytes required trimming by ERAP1 from NH_2_-terminally elongated precursors and was much dependent on the inflammatory proteasome induced by IFN-γ (91). This reflects that tissue-specific versions of the proteasome as well as inflammatory conditions may affect the immunogenicity of the identified epitopes through differential processing of the parental protein (92). Similarly, in the COS-7 cells used for presentation, an immunologically dominant epitope for the immune response against influenza virus was not generated from the parental protein, although the influenza peptide-specific TCR originated from an influenza-infected patient (80). Accordingly, the identification of self-peptides as ligands of autoreactive TCRs is not sufficient to establish a role as an epitope for the autoimmune response. The immunogenicity must be verified within the parental protein and in the target cell, unless the peptide is detected in the respective immunopeptidome.

### 3.7 Identification of self-epitopes of HLA-class II-restricted autoimmune responses

Two principle situations need to be considered for the identification of epitopes of autoreactive CD4^+^ T cells: the CD4^+^ T cells may have a direct role in the inflammatory infiltrate or control autoantibody production. In principle, HLA-class II-restricted TCRs from CD4^+^ cells are subject to the same conditions in terms of polyspecificity and TCR recognition motif as CD8^+^ T cells: They are ligated by immunogenic peptides with specific amino acid patterns defined by the anchor and TCR contact residues. Unlike MHC-class I molecules, MHC-class II has an open peptide-binding groove, allowing easy peptide linkage regardless of a given peptide length. For TCRs with known specificity and MHC restriction, the recognition motif of mimotopes isolated from peptide libraries matched the actual antigen originally used to expand the T-cell clones from which the TCRs studied were derived (70). Birnbaum et al. coupled TCRs of known specificity to streptavidin-coated magnetic beads and used them to select yeast-displayed pMHC libraries carrying the cognate MHC-class II molecule with bound peptides by magnetic enrichment. Searching with the TCR recognition motif of a myelin-basic protein (MBP)-specific TCR for proteins with homologous peptide sequences in databases revealed several ligands, including the actual immunogenic MBP.

Still, the first step is to define the restricting HLA-class II molecule for the TCRs of interest. This may be challenging because immunogenic molecules are engulfed by APCs from the extracellular fluid or introduced into the antigen processing and presentation pathway from cytosolic proteins or intracellular organelles by autophagy. Thus, autoimmune responses may lack a target cell for identification of the restricting HLA-class II allotype. A recent study in the search for HLA-class II-presented tumor antigens circumvented this problem by generating peptide libraries from fragmented tumor cDNA shorter than 150base pairs, encoding peptides of 50 amino acids or less that should cover most of the mRNA-derived peptides. Exposure to TCR hybridomas generated from tumor-infiltrating CD4^+^ T cells identified several reporter clones that carried a new tumor-specific antigen (93). However, knowing the target cell was crucial for the identification of the T cell epitopes also in this approach.

For extracellular autoantigens, identification of immunogenic self-peptides of pathogenic CD4^+^ T cells may be difficult, because the source of antigens is not defined, and MHC restriction cannot be established due to a lacking target cell. Therefore, in a clinical setting, all HLA-class II alleles from a single patient must be considered when analyzing a peptide library. The lack of a target cell also confounds proof as an immunogenic self-peptide of the autoimmune response by knock down or knock out.

As another issue, the pathogenic CD4^+^ T cells do not necessarily have to be contained in the inflammatory infiltrate and are then not reliably accessible for TCRs identification. While in generalized pustular psoriasis the association with HLA-DRB1*14, HLA-DQB1*05, and HLA-DQB1*03 as well as strong CD4^+^ T cell clonality suggested a pathogenic role of CD4^+^ T cells in the inflammatory infiltrate (94), the CD4^+^ T cells controlling autoantibody formation may be present in the lymph node, where dendritic cells potentially present self-molecules to T cells. Furthermore, exogenous antigens may have activated the CD4^+^ T cells controlling autoantibody formation: The production of pathogenic Desmoglein 1 autoantibodies bei *Fogo selvagem*, the HLA-class-II-associated endemic form of pemphigus foliaceus in South America, can be induced by a cross-reactive immune response against the protein antigen LJM11 of the salivary glands of the sandfly (*Lutzomyia longipalpis*), which is presented by the disease-associated HLA-class II-molecules (95, 96). An approach in a humoral autoimmune response may be to screen the autoantigens defined by the autoantibodies for the TCR recognition motif. Accordingly, an increased frequency of CD4^+^ T cells with specificity for bullous pemphigoid antigen 2 (BPAG2) was observed in patients with bullous pemphigoid, an HLA-class II-associated blistering skin disease mediated by antibodies against BPAG2 (97, 98). Each strategy to identify potential HLA-class II-presented T-cell epitopes of autoimmune responses must therefore be designed separately according to the particular circumstances.

## 4 Conclusions

Despite major methodological advances, the identification of immunogenic self-peptides of the immunopeptidome is complex and requires multilayered methodological approaches. After the identification of the pathogenic, clonally expanded TCRs, the HLA allotype restricting the autoimmune response must first be determined for these TCRs. As with psoriasis, this may be the disease-associated HLA allele. The concept that HLA-class I-restricted autoimmune responses are most likely directed against a specific target cell expressing the parental protein of the immunogenic self-peptide may be a key to identify the immunogenic self-peptides. It allows to determine the HLA restriction of TCRs and limits the potential T-cell epitopes to the transcriptome or immunopeptidome of the target cell. The determination of the recognition motif of the pathogenic TCRs using peptide libraries allows the search for proteins with homologous peptide sequences. In the case of nonlinearly encoded peptides, the immunogenic peptides can only be identified in the respective immunopeptidome. The significance of the candidate peptides identified in this way as actual targets of the autoimmune response must then be verified under the natural conditions of processing and presentation from the parental protein. Only then is the process of identifying immunogenic self-peptides in autoimmune diseases complete.

## Author contributions

The author confirms being the sole contributor of this work and has approved it for publication.
